# Prostate fibroblasts and prostate cancer associated fibroblasts exhibit different metabolic, matrix degradation and PD-L1 expression responses to hypoxia

**DOI:** 10.3389/fmolb.2024.1354076

**Published:** 2024-03-22

**Authors:** Jesus Pacheco-Torres, Raj Kumar Sharma, Yelena Mironchik, Flonne Wildes, W. Nathaniel Brennen, Dmitri Artemov, Balaji Krishnamachary, Zaver M. Bhujwalla

**Affiliations:** ^1^ Division of Cancer Imaging Research, The Russell H. Morgan Department of Radiology and Radiological Science, The Johns Hopkins University School of Medicine, Baltimore, MD, United States; ^2^ Instituto de Investigaciones Biomédicas Sols-Morreale, CSIC, Madrid, Spain; ^3^ Sidney Kimmel Comprehensive Cancer Center, The Johns Hopkins University School of Medicine, Baltimore, MD, United States; ^4^ Radiation Oncology and Molecular Radiation Sciences, The Johns Hopkins University School of Medicine, Baltimore, MD, United States

**Keywords:** fibroblast, hypoxia, metabolism, prostate cancer, cancer associated fibroblast, PD-L1

## Abstract

Fibroblasts are versatile cells that play a major role in wound healing by synthesizing and remodeling the extracellular matrix (ECM). In cancers, fibroblasts play an expanded role in tumor progression and dissemination, immunosuppression, and metabolic support of cancer cells. In prostate cancer (PCa), fibroblasts have been shown to induce growth and increase metastatic potential. To further understand differences in the functions of human PCa associated fibroblasts (PCAFs) compared to normal prostate fibroblasts (PFs), we investigated the metabolic profile and ECM degradation characteristics of PFs and PCAFs using a magnetic resonance imaging and spectroscopy compatible intact cell perfusion assay. To further understand how PFs and PCAFs respond to hypoxic tumor microenvironments that are often observed in PCa, we characterized the effects of hypoxia on PF and PCAF metabolism, invasion and PD-L1 expression. We found that under normoxia, PCAFs displayed decreased ECM degradation compared to PFs. Under hypoxia, ECM degradation by PFs increased, whereas PCAFs exhibited decreased ECM degradation. Under both normoxia and hypoxia, PCAFs and PFs showed significantly different metabolic profiles. PD-L1 expression was intrinsically higher in PCAFs compared to PFs. Under hypoxia, PD-L1 expression increased in PCAFs but not in PFs. Our data suggest that PCAFs may not directly induce ECM degradation to assist in tumor dissemination, but may instead create an immune suppressive tumor microenvironment that further increases under hypoxic conditions. Our data identify the intrinsic metabolic, ECM degradation and PD-L1 expression differences between PCAFs and PFs under normoxia and hypoxia that may provide novel targets in PCa treatment.

## Introduction

Prostate cancer (PCa) is the most commonly diagnosed cancer in men, and the second cause of cancer-related mortality, accounting for approximately 11% of death caused by cancer among men in the US (Siegel et al., 2022). Most PCa-related deaths are due to advanced metastatic disease. Although the overall PCa incidence has remained stable during the last decade, the incidence of metastatic PCa has increased from 4% to 8% over this time (Siegel et al., 2022). Furthermore, despite recent advances in cancer therapies, PCa mortality rates have not declined, highlighting the importance of developing therapeutic alternatives. The successes with immune checkpoint inhibitor treatments observed in some cancers have also not been replicated in PCa (Noori et al., 2023).

An expanded understanding of the facilitators of PCa cell invasion and immune suppression can provide novel targets in preventing PCa progression and metastasis (Brennen et al., 2016; Krueger et al., 2019; Brennen et al., 2021). Fibroblasts are one of the most ubiquitous cells in connective tissue, playing a crucial role in extracellular matrix (ECM) synthesis and remodeling, collagen production and wound healing (Chen et al., 2021). Cancer-associated fibroblasts (CAFs), are often found in primary and metastatic cancers (Gong et al., 2020; Biffi and Tuveson, 2021; Li et al., 2021). Although all fibroblast share some common characteristics, they also exhibit high molecular and functional heterogeneity in both normal and tumoral tissues (Biffi and Tuveson, 2021). While normal fibroblasts can inhibit cancer progression (Trimboli et al., 2009; Alkasalias et al., 2014), CAFs promote progression by remodeling the ECM (Gaggioli et al., 2007; Goetz et al., 2011), modulating cancer cell metabolism (Zhao et al., 2016; Sherman et al., 2017), establishing an immunosuppressive TME (Kraman et al., 2010; Feig et al., 2013; Lakins et al., 2018), conferring therapy resistance (Hessmann et al., 2018; Vennin et al., 2019; Cheteh et al., 2017), promoting tumor invasion and metastasis (Gaggioli et al., 2007; Grugan et al., 2010; Goetz et al., 2011; Vennin et al., 2019), secreting pro-tumorigenic ligands (Orimo et al., 2005; Grugan et al., 2010), and providing metabolites (Pavlides et al., 2009; Sousa et al., 2016; Olivares et al., 2017) and lipids (Auciello et al., 2019; Gong et al., 2020) as substrates for cancer cells.

In PCa, CAFs have been associated with progression to metastatic disease and therapeutic resistance (Bedeschi et al., 2023). Recently, two PCa CAF subtypes were identified (Pan et al., 2023), one of which was associated with poor prognosis and resistance to immune checkpoint inhibitor treatment. CAF targeting strategies have, however, met with limited success (Chen et al., 2021), highlighting the importance of expanding our understanding of CAFs as well as normal fibroblasts. Similar to CAFs, hypoxia contributes to aggressiveness, resistance to treatment, and metastatic dissemination (Semenza, 2010; Penet et al., 2011; Gilkes and Semenza, 2013; Gilkes et al., 2014). PCa ranks high among the malignancies in which hypoxia plays a major role in treatment resistance and metastases (Fraga et al., 2015). Understanding the metabolic, invasion and immune checkpoint response of PCAFs and prostate fibroblasts to hypoxia is, however, relatively unexplored, and can provide new insights into effective treatment for PCa. The PD-L1/PD-1 immune checkpoint axis is being increasingly exploited in the effective treatment of several cancers (Sun et al., 2018) but has had limited success in PCa (Picardo and Hansen, 2019). The role of CAFs in the PD-L1/PD-1 axis of PCa, within the context of hypoxia, can provide insights into the limited success of immune checkpoint inhibition in PCa.

Here, for the first time, we investigated the metabolic profile, invasiveness and ECM degradation of normal human PFs compared to human PCAFs under normoxia and hypoxia. These studies were performed using a magnetic resonance (MR) compatible cell perfusion system that allowed dynamic quantitation of invasion and degradation. Quantitative metabolic characterization was performed with high resolution 1H MR spectroscopy of cell extracts. We characterized expression of the immune checkpoint PD-L1 in PFs and PCAFs under normoxia and hypoxia. We also established the expression of PD-L1 by CAFs in PC-3 human prostate cancer (hPCa) xenografts and in hPCa tissue in a human tissue microarray (TMA). Our data identified significant differences between PF and PCAF metabolism and the ability to invade and degrade ECM under normoxia and hypoxia. Significant differences in PD-L1 expression were also identified in PFs and PCAFs under normoxia and under hypoxia.

## Materials and methods

### Cells and cell culture conditions

Experiments were performed using normal human PFs (WPMY-1, ATCC, Manassas, VA) and human PCAFs (Asterand Bioscience, Detroit, MI). WPMY-1 were derived from stromal cells from the peripheral zone of a histologically normal adult prostate (Webber et al., 1999). PCAFs were obtained from an adenocarcinoma of the prostate gland. WPMY-1 and PCAFs were cultured in DMEM supplemented with 5% and 10% fetal bovine serum (FBS) respectively. Cells were maintained in a humidified atmosphere at oxygen concentration [O_2_] ≥ 20%, and 5% CO_2_ at 37 °C for 48 h. To achieve hypoxia, cells were maintained at [O_2_] < 1%, and 5% CO_2_ at 37 °C for 48 h.

### Cell perfusion system and MR data acquisition

For each intact cell perfusion experiment, 1 × 10^6^ WMPY-1 or 3 × 10^6^ PCAFs were seeded per 0.5 mL of Plastic Plus beads (SoloHill Engineering, Ann Arbor, MI, USA) in multiple dishes, and allowed to grow for 3 days or 6 days, respectively, to achieve sufficient cell density. An in-house customized chamber containing Matrigel^®^ at a concentration of 8.8 mg/mL was used to determine the degradation of ECM by the cells. Fibroblast covered beads were layered on the ECM gel chamber in a customized 10 mm MR tube. The oxygen concentration in the sample was kept at [O_2_] ≥ 20% during normoxia, and adjusted to [O_2_] ≤ 1% during hypoxia experiments. A layer of perfluorocarbon doped alginate beads was interspersed within the layers of the cells to monitor the oxygen tension in the sample using ^19^F MR relaxometry. A detailed description of the MR cell perfusion system has been previously published (Pilatus et al., 2000; Shah et al., 2012). An inversion recovery ^19^F sequence, spatially localized within the perfluorocarbon layer, provided T_1_ relaxation rates of the embedded perfluorocarbons, that reported on oxygen concentrations. The temperature was maintained at 37 °C and the pH at 7.30 ± 0.15 for all MR experiments.

The following series of MR acquisitions were obtained on a 9.4 T MR spectrometer (Bruker, Billerica, MA, USA) every 12 h over a period of 48 h. Proton MRI was performed to evaluate the overall sample preparation, to visualize the geometry of the ECM gel, and to detect changes in the integrity of the ECM gel due to invasion and degradation by fibroblasts. Two-dimensional images were acquired using a spin-echo imaging sequence with a field of view (FOV) of 40 mm, repetition time (TR) = 1 s, and echo time (TE) = 30 m from a 2 mm thick central slice of the sample. Degradation of the ECM by fibroblasts was determined at multiple time points relative to the initial time point from the proton images. The extent of ECM degradation was estimated by drawing a region of interest (ROI) around the ECM gel region using NIH ImageJ software. The degradation index t) at time t was defined using Eq. 1:

$$Degradation\;index\;\left(t\right)=\frac{{ROI}_{t0}-{ROI}_{t}}{{ROI}_{t0}}$$ (1)

where *t* is the time point in hours, *t0* is the initial time point, *ROI*_*t0*_ and *ROI*_*t*_ are the areas of the ROI at time points *t0* and *t*, respectively. t0 refers to the first imaging time point, which was approximately 2 h after the fibroblasts were placed in contact with the ECM.

One-dimensional (1D) ^1^H MR profiles of intracellular water with a spatial resolution of 62.5 μm were acquired along the length (*z*-axis) of the sample by diffusion-weighted (DW) 1D ^1^H magnetic resonance imaging (MRI), using gradient pulses of 3 m duration with 18 G/cm gradient strength and employing a diffusion weighting time of 100 ms. These profiles were used to derive an invasion index by quantifying the number of cells invading the ECM, since signal from slow-diffusing water, which represents intracellular water, is directly proportional to the number of cells (Pilatus et al., 1997). The invasion index t) at time t was calculated using Eq. 2:

$$Invasion\;index\;\left(t\right)=\frac{I_{p,7mm\left(t\right)}}{I_{p\left(t\right)}}-\frac{I_{p,7mm\left(t0\right)}}{I_{p\left(t0\right)}}$$ (2)

where *I*_*p,7mm(t)*_ and *I*_*p,7mm(t0)*_ are the integral values of the signal at time t and t0 respectively, obtained by integrating the intracellular water signal over a 7 mm region starting at the base of the ECM chamber, and *I*_*p(t)*_ and *I*_*p(t0)*_ are the integrals of the profile of the entire sample at times t and t0, respectively.

## Dual-phase extraction and high-resolution ^1^H MRS

High-resolution ^1^H MR spectra of fibroblasts, maintained under normoxia or hypoxia, were obtained to characterize metabolic differences between PFs and PCAFs. Approximately 1 × 10^7^ WPMY-1 and PCAFs were incubated for 48 h under normoxia or hypoxia, as detailed earlier. Water-soluble and lipid fractions were extracted from the fibroblasts using a dual-phase extraction method (Bharti et al., 2018). Briefly, pelleted fibroblasts were washed with ice-cold saline, then mixed with 4 mL of ice-cold methanol and vigorously vortexed. After keeping samples on ice for 15 min, 4 mL of chloroform were added, vortexed vigorously and kept on ice for an additional 10 min. Finally, 4 mL of water were added and the samples were vortexed again. All procedures were performed on ice and samples were stored at 4°C overnight for phase separation and then centrifuged at 15,000 × *g* at 4°C for 30 min. The aqueous phase containing water-soluble metabolites was collected (Tkac et al., 1999). Methanol in the aqueous phase was first evaporated under nitrogen gas, and any water remaining in the aqueous phase was lyophilized. Dried aqueous phase extracts were re-suspended in 0.6 mL deuterated water (D_2_O) for magnetic resonance spectroscopy (MRS) analysis. 3-(trimethylsilyl) propionic 2,2,3,3-d4 acid sodium salt (TSP) dissolved in D_2_O was used as an internal standard. Lipid phase extracts were dried under nitrogen gas stream and re-suspended in 0.6 mL deuterated chloroform and methanol in a 2:1 ratio containing tetramethylsilane (TMS) 0.05% v/v.

High-resolution ^1^H MR spectra were acquired on a Bruker Biospin Avance-III 750 MHz NMR (Bruker Biospin Billerica, MA, USA) spectrometer operating at a proton frequency of 750.21 MHz using a 5-mm broad band inverse (BBI) probe equipped with z-gradient accessories. For quantitative analysis of metabolites, integrals of resonances were determined and normalized to the number of cells and compared to the TSP standard (aqueous phase) or TMS standard (lipid phase) to obtain relative concentrations. Spectra were analyzed using MNova software (Mestrelab Research, Santiago de Compostela, Spain). Each experiment was carried out at least 4 times: WPMY-1, n = 8 for normoxia and n = 6 for hypoxia; PCAF, n = 4 for normoxia and n = 5 for hypoxia.

To identify the commonality and differences in metabolism in PFs and PCAFs under normoxia and hypoxia, Venn diagrams were created using Venny 2.1 online program provided by BioinfoGP (https://bioinfogp.cnb.csic.es/tools/venny/).

To identify the molecular pathways impacted by the significant metabolic differences between the groups, we used the publicly available database ConsensusPathDB (http://cpdb.molgen.mpg.de). Pathways with q-values <0.001 were considered significant. Only those pathways for which 25% or more of the total metabolites were significantly depleted are presented for the ConsensusPathDB analysis.

### Invasion assay

Cell invasion was determined by the Cultrex^®^ Basement Membrane Extract Cell Invasion Assay (Catalog# 3455–024-K, Trevigen, Gaithersburg, MD, USA) following the manufacturer’s instructions with minor modifications. Briefly, 1 × 10^5^ fibroblasts were loaded in the upper chamber of a 24-well transwell plate while DMEM medium with 10% FBS was present in the lower chamber. Fibroblasts were incubated for 24 and 48 h under normoxic or hypoxic conditions. Cell dissociation solution with 1 µM of Calcein-AM was used to collect fibroblasts after incubation. The plate was read using 485 nm excitation and 520 nm emission filters. Standard curves established for WMPY-1 and PCAFs were used to calculate percentages of invaded fibroblasts.

### RNA isolation, cDNA synthesis, RT-PCR, immunoblotting

For mRNA expression studies, approximately 0.4 × 10^6^ WPMY-1 or PCAFs were seeded in 100 mm Petri dishes. Twenty-4 hours later, cells were subjected to hypoxia for 24 h by placing them in a modular incubator chamber (Billups-Rothenberg, Del Mar, CA), flushed at 2 p. s.i. for 3 min with a gas mixture of 0.2% O2, 5% CO_2_, and balance N_2_. Total RNA was isolated from cells using the QIAshredder and RNeasy Mini kit (Qiagen, Valencia, CA, USA) as per the manufacturer’s protocol. cDNA was prepared using the iScript cDNA synthesis kit (Bio-Rad, Hercules, CA, USA). Real-time PCR of cDNA samples was performed using IQ SYBR Green supermix and gene specific primers in the iCycler real-time PCR detection system (Bio-Rad). All primers were designed using either Beacon designer software 7.8 (Premier Biosoft, Palo Alto, CA, United States) or publicly available Primer3plus software. The expression of target RNA relative to the housekeeping gene hypoxanthine phosphoribosyltransferase 1 (HPRT1) was calculated based on the threshold cycle (C_t_) as R = 2-^Δ(ΔCt)^, where ΔC_t_ = C_t_ of target gene - C_t_ of HPRT1 and Δ(ΔC_t_) = ΔC_t_ siRNA treated cells - ΔC_t_ untreated cells.

For immunoblot studies, 1.5 × 10^6^ cells were plated in 100 mm Petri dishes. As mentioned above, 24 h later, cells were subjected to hypoxia for 48 h or maintained under normoxic condition. Total protein from cells was extracted with a radio-immunoprecipitation assay (RIPA) buffer with various protease and phosphatase inhibitors to prevent degradation of the protein. 7.5% SDS-PAGE gel was used for protein electrophoresis and immunoblot analysis. A monoclonal primary antibody against human PD-L1 (1: 1000, GeneTex, Cat. No. GTX104763, Irvine, CA), and an anti-GAPDH antibody (mouse monoclonal, Sigma, St. Louis, MO) were used. Immunoblots were developed using SuperSignal™ West Pico PLUS Chemiluminescent Substrate kit (ThermoFisher Scientific) following manufacturer’s instructions.

### Tumor immunohistochemistry

PC-3 tumors derived from human PC-3 cells inoculated subcutaneously in the flank of 4–6 weeks old severe combined immunodeficient (SCID) male mice were excised from euthanized mice at volumes of ∼300–400 mm^3^, and formalin fixed for PD-L1 immunostaining. Animal studies were conducted under an approved Animal Care and Use Committee protocol.

A human prostate cancer (hPCa) tissue microarray (TMA) with 5 µm thick, 1.5 mm diameter tissue sections (PR243D) was purchased from Tissue Array (Derwood, MD, USA) and immunostained for PD-L1.

For PD-L1 immunostaining, tumor sections were deparaffinized and rehydrated in gradient alcohol. Antigen retrieval was achieved by boiling the slides in pre-warmed citrate buffer, pH 6.0 solution for 20 min. Peroxidase blocking, serum free protein blocking, 1% BSA and normal goat serum blocking were performed on slides prior to overnight incubation at 4°C with a PD-L1 antibody (Proteintech, Cat. #-17952-1, Rosemount, IL, 1:400 dilution). Horseradish peroxidase conjugated secondary antibody (Vector Laboratories, Burlingame, CA) was used to recognize the primary antibody. After incubation with the secondary antibody for 1h, DAB (3,3′-diaminobenzidine, Epredia, Kalamazoo, MI) chromogen was used to develop color, following which slides were counter stained with hematoxylin (Vector Laboratories). Stained tissue slides were scanned with an Aperio ScanScope XT slide scanner (Aperio Technologies, Vista, CA).

### Statistical analysis

Statistical analyses were performed using GraphPad Prism 4 software (GraphPad Software, Inc., San Diego, CA, USA, RRID:SCR_002798). To determine the statistical significance of the quantified data, an unpaired two-tailed Student’s t-test was performed. *p* values ≤0.05 were considered significant unless otherwise stated.

## Results

### Cell invasion and ECM degradation

**FIGURE 1:**
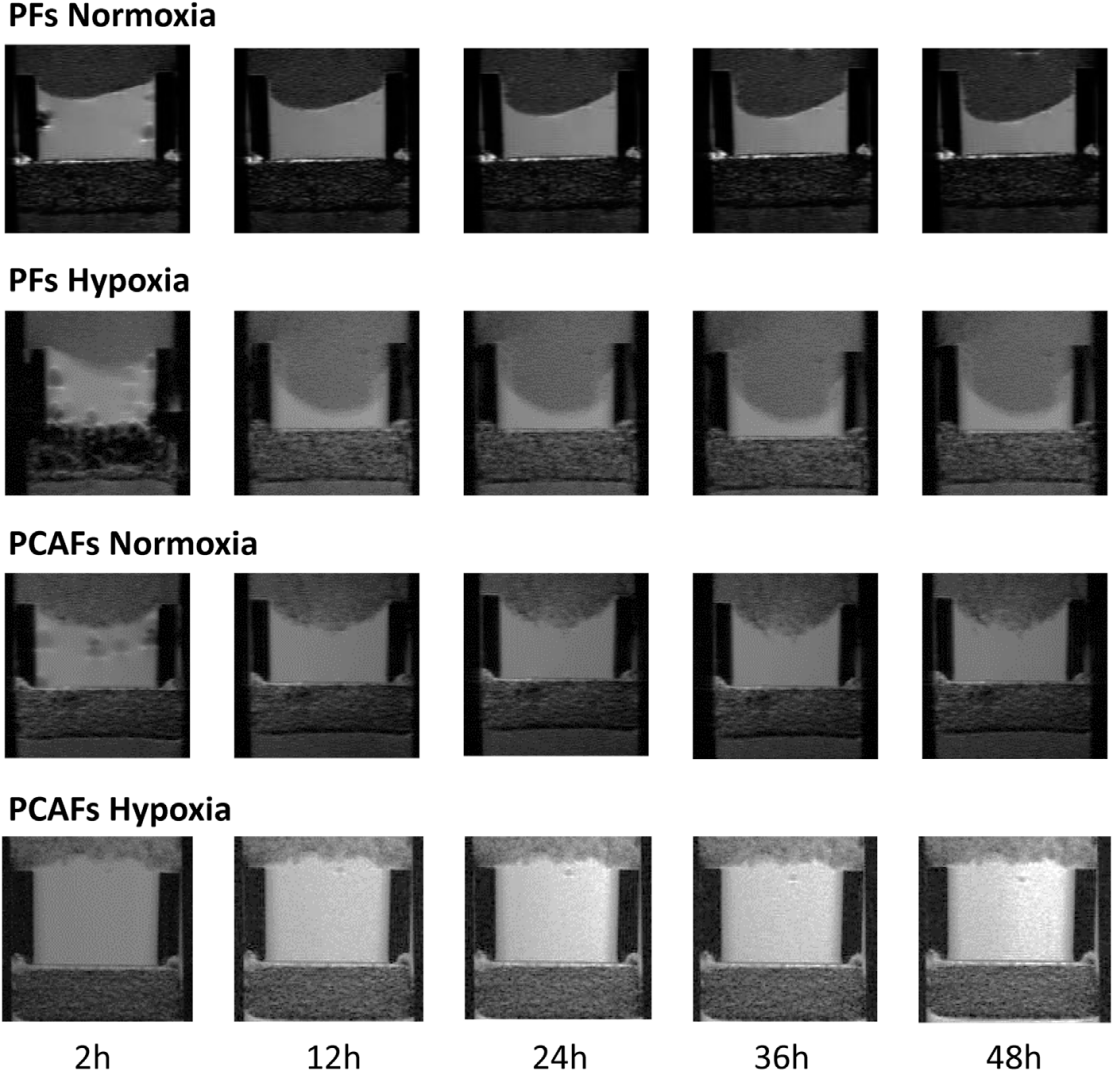
Representative T1-weighted ^1^H MR images displaying the region of the ECM chamber to demonstrate degradation of the ECM by PFs and PCAFs under normoxia and hypoxia over a period of 48 h.

Representative ^1^H MR images in Figure 1 display ECM gel degradation by PFs (WMPY-1) and PCAFs under normoxic and hypoxic conditions at multiple time points. PFs degraded the ECM under both conditions, with significantly increased ECM degradation under hypoxia. Under normoxia, PCAFs also degraded ECM with a small but significant decrease compared to PFs. In the presence of hypoxia, however, ECM degradation by PCAFs was significantly reduced.

**FIGURE 2:**
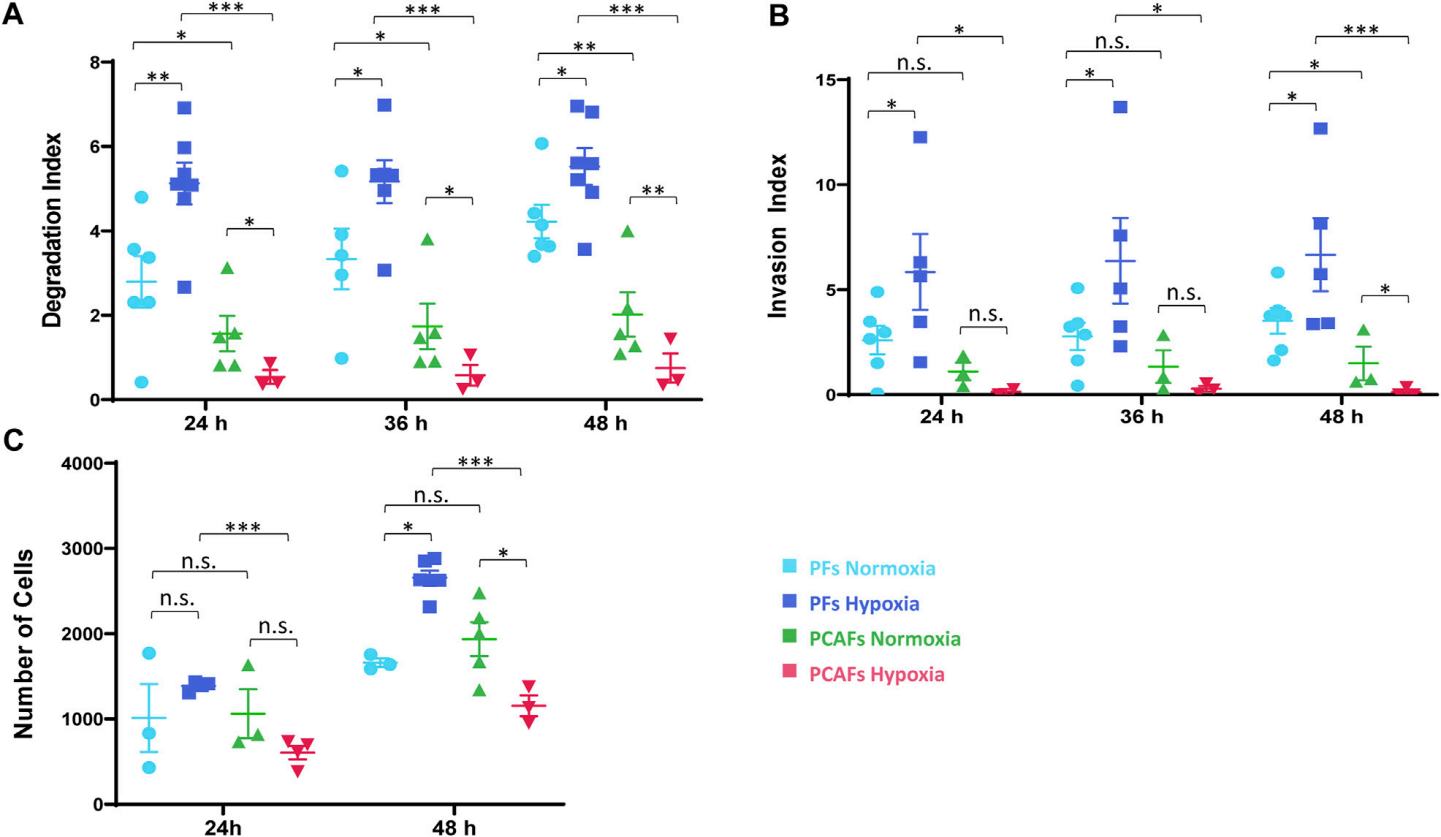
**(A)** Degradation index estimated from T1 images at 24 and 48 h relative to the initial time point. **(B)** Invasion index estimated from intracellular water profiles at 24 and 48 h relative to the initial time point. **(C)** Invasion assay of PFs and PCAFs at 24 and 48 h under normoxia and hypoxia. *p < 0.05, **p < 0.01, ***p < 0.001. Values represent Mean ± SE.

Changes in ECM invasion and degradation indices of PFs and PCAFs under normoxia and hypoxia are presented in Supplementary Figure S1. Quantitative degradation and invasion indices at 24, 36 and 48 h are summarized in Figures 2A, B, respectively. PCAFs displayed significantly lower degradation compared to PFs under both normoxia and hypoxia at all time points. PFs were significantly more invasive than PCAFs under hypoxia at all time points. Under normoxia, PFs were significantly more invasive than PCAFs by 48 h. Hypoxia significantly increased PF degradation and invasion compared to normoxia at all time points. For PCAFs, hypoxia significantly decreased ECM degradation at all time points, and ECM invasion at 48 h compared to normoxia.

A cell invasion assay independently confirmed the MR cell perfusion results, as shown in Figure 2C. Hypoxia reduced PCAF invasion but increased PF invasion by 48 h, as evident from the reduced number of cells that invaded through the basement membrane.

### Metabolic characterization of PFs and PCAFs under normoxia and hypoxia

**FIGURE 3:**
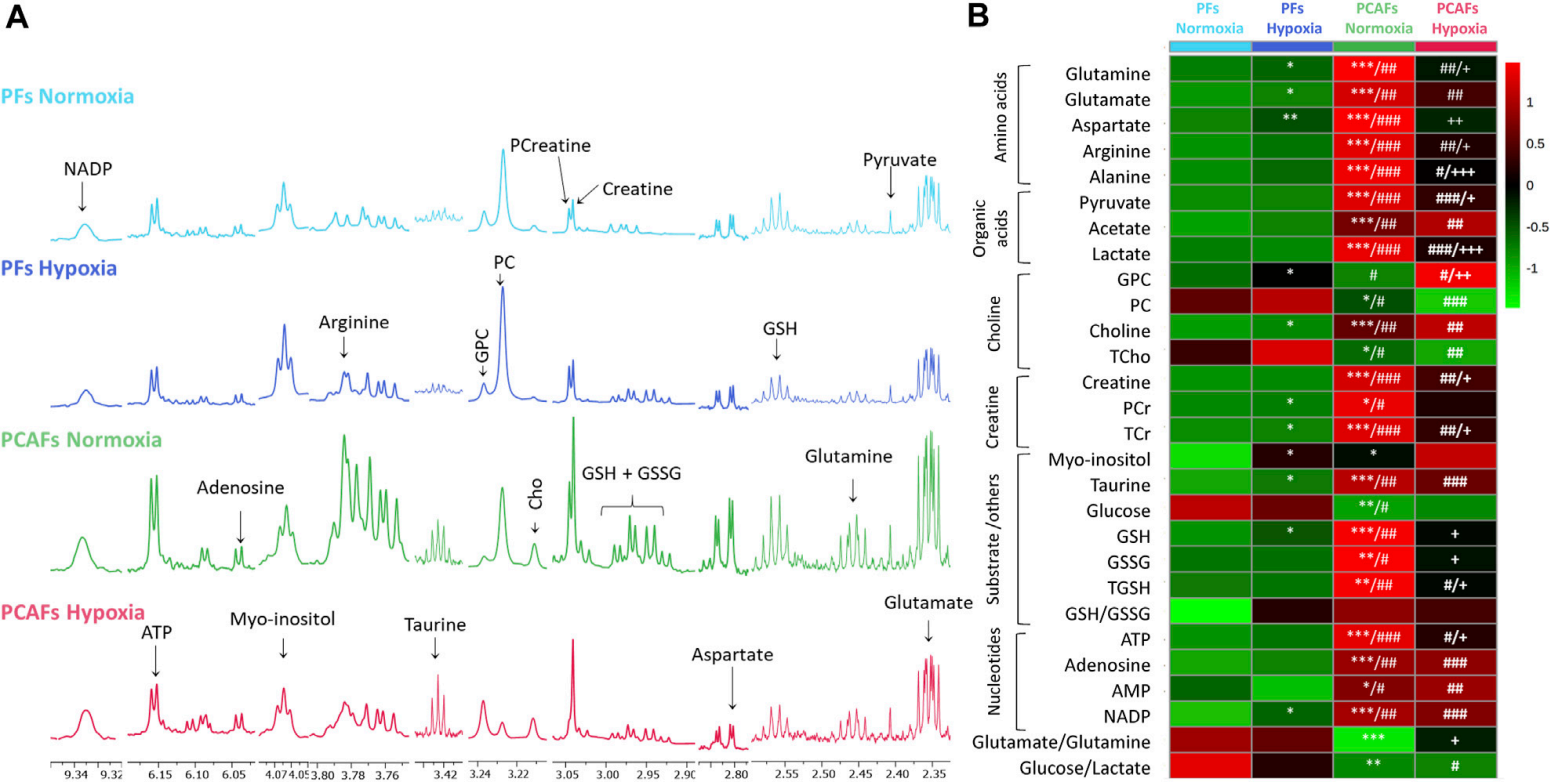
**(A)** Representative high-resolution ^1^H MR spectra obtained from the aqueous phase of PFs incubated for 48 h under normoxia (light blue), hypoxia (dark blue), and PCAFs incubated for 48 h under normoxia (green) and hypoxia (red). All spectra were plotted on the same vertical scale and acquired with identical experimental parameters. **(B)** Metabolic heat map, generated from quantitative analysis of high-resolution ^1^H MR spectral data of the aqueous phase, displaying differences in the metabolic profile of PFs and PCAFs incubated under normoxia and hypoxia. Heat maps were created using the MetaboAnalyst platform (www.metaboanalyst.ca) to visualize the metabolic patterns. Each heat map represents the average of three to six replicates per group. The integral area under the peak was normalized to the number of cells for each sample. TSP dissolved in D_2_O was used as a quantitative reference in the spectral analysis. GPC, glycerophosphocholine; PC, phosphocholine; Cho, free choline; TCho, total choline signal consisting of Cho, PC and GPC; PCr, phosphocreatine; TCr, total creatine signal consisting of Creatine and PCr; GSH, glutathione; GSSG, oxidized glutathione; TGHS, total glutathione signal consisting of GSH and GSSG. *p ≤ 0.05, **p ≤ 0.01, ***p ≤ 0.001, compared to PFs normoxia, ^#^ *p* ≤ 0.05, ^##^ *p* ≤ 0.01, ^###^ *p* ≤ 0.001, compared to PFs hypoxia, ^+^ *p* ≤ 0.05, ^++^ *p* ≤ 0.01, ^+++^ *p* ≤ 0.001, compared to PCAF normoxia.

**FIGURE 4:**
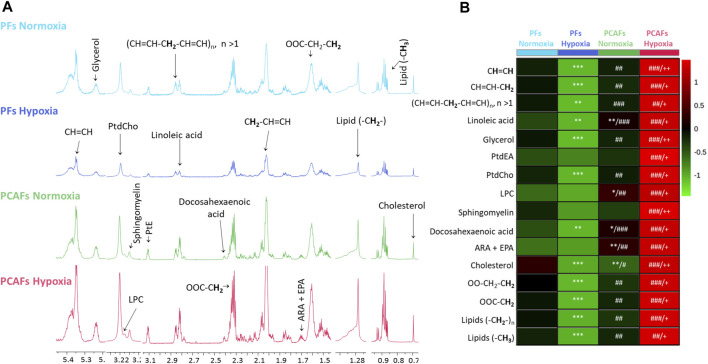
**(A)** Representative high-resolution ^1^H MR spectra obtained from the lipid phase of PFs incubated for 48 h under normoxia (light blue), hypoxia (dark blue), and PCAFs incubated during 48 h under normoxia (green) and hypoxia (red). All spectra were plotted on the same vertical scale and acquired with identical experimental parameters. **(B)** Metabolic heat map, generated from quantitative analysis of high-resolution ^1^H MR spectral data of the lipid phase, displaying differences in the metabolic profile of PFs and PCAFs incubated under normoxia and hypoxia. Heat maps were created using the MetaboAnalyst platform (www.metaboanalyst.ca) to visualize the metabolic patterns. Each heat map represents the average of three to six replicates per group. The integral area under the peak was normalized to the number of cells for each sample. Lipids (-CH_3_), methyl groups of fatty acids; Lipids (-CH_2_-), methylene groups of fatty acids; OOC-C**H**_**2**_, methylene groups at the α position of the carboxylic function; OOC-CH_2_-C**H**_**2**_, methylene groups at the β position of the carboxylic function; ARA, arachidonic acid; EPA, eicosapentaenoic acid; PtdEA, phosphatidylethanolamine; LPC, lysophosphatidylcholine; PtdCholine, phosphatidylcholine; (CH = CH-C**H**_**2**_-CH = CH)n, diallylic methylene protons; CH = CH-C**H**_**2**_, methylene groups at the α position of a double bond; C**H** = C**H**, fatty acid double bonds. *p ≤ 0.05, **p ≤ 0.01, ***p ≤ 0.001, compared to PF normoxia, ^#^ *p* ≤ 0.05, ^##^ *p* ≤ 0.01, ^###^ *p* ≤ 0.001, compared to PF hypoxia, ^+^ *p* ≤ 0.05, ^++^ *p* ≤ 0.01, ^+++^ *p* ≤ 0.001, compared to PCAF normoxia.

**FIGURE 5:**
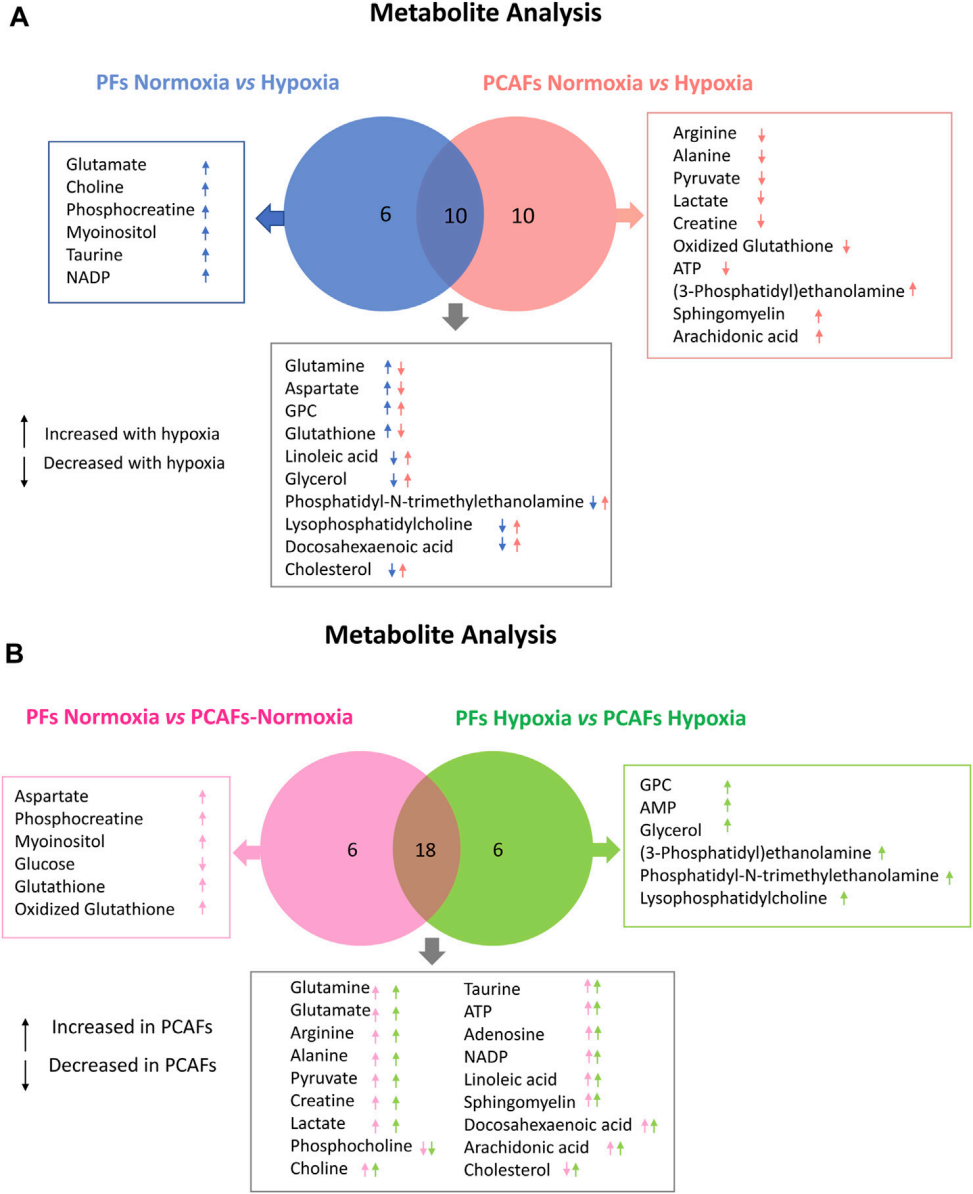
Venn Diagrams displaying commonalities and differences. **(A)** Comparison of significant changes in metabolites between PFs under normoxia and hypoxia and PCAFs under normoxia and hypoxia. **(B)** Comparison of significant changes in metabolites between PFs and PCAFs under normoxia, and PFs and PCAFs under hypoxia. All metabolites listed were significantly different (*p* ≤ 0.05).

Representative ^1^H MR spectra from the water and lipid fractions of PFs and PCAFs incubated under normoxia and hypoxia for 48 h are presented in Figures 3A, 4A Supplementary Figure S4, respectively, together with the corresponding heat maps of the quantified metabolites in Figures 3B, 4B respectively. Quantified metabolic data are presented in Table 1 for the water-soluble fraction, and in Table 2 for the lipid fraction of both cell lines. Venn diagrams of aqueous and lipid-phase metabolites displaying differences and commonalities for comparisons between normoxia and hypoxia within each cell line and across the 2 cell lines are presented in Figures 5A, B. The corresponding pathway analyses are presented in the Venn diagrams in Supplementary Figures 2A, B.

**TABLE 1:** Water-soluble metabolite concentrations (mM/cell) in PFs and PCAFs corresponding to Figure 3. Values were generated from the quantitative analysis of high-resolution ^1^H MR spectra obtained from the aqueous phase of PFs and PCAFs maintained for 48 h under normoxia or under 1% O_2_ (hypoxia). Values represent Mean ± SEM from five to seven independent experiments. GPC: glycerophosphocholine, PC: phosphocholine, TCholine: Total choline (Choline + PC + GPC), PCreatine: phosphocreatine, TCreatine: Total Creatine (Creatine + PCreatine), GSH: reduced glutathione, GSSG: oxidized glutathione, TGSH: total glutathione (GSH + GSSG). *p < 0.05, **p < 0.01, ***p < 0.001 PF normoxia vs. PF hypoxia;+*p*< 0.05, ^++^ *p* < 0.01, ^+++^ *p* < 0.001 PCAF normoxia vs*.* PCAF hypoxia;^#^ *p* < 0.05,^##^ *p* < 0.01,^###^ *p* < 0.001 PF normoxia vs*.* PCAF normoxia;^x^ *p* < 0.05, ^xx^ *p* < 0.01, ^xxx^ *p* < 0.001 PF hypoxia vs*.* PCAF hypoxia.

Metabolites	PF normoxia	PF hypoxia	PCAF normoxia	PCAF hypoxia
Glutamine	1.8 ± 0.3	3.6 ± 0.6*	32 ± 6^###^	9 ± 1^+^/^xx^
Glutamate	15 ± 1	19 ± 1*	99 ± 17^###^	65 ± 11^xx^
Aspartate	2.5 ± 0.2	3.6 ± 0.3**	11 ± 1^###^	4.5 ± 0.5^++^
Arginine	6.6 ± 0.6	9 ± 1	32 ± 5^###^	19 ± 2^+^/^xx^
Alanine	2.3 ± 0.3	3.3 ± 0.6	14.4 ± 0.5^###^	7 ± 1^+++^/^x^
Pyruvate	0.28 ± 0.04	0.32 ± 0.05	1.6 ± 0.2^###^	0.99 ± 0.08^+^/^xxx^
Acetate	0.6 ± 0.1	0.7 ± 0.2	2.0 ± 0.2^###^	2.1 ± 0.3^xx^
Lactate	14 ± 2	14 ± 2	54 ± 4^###^	34.1 ± 0.9^+++^/^xxx^
GPC	1.30 ± 0.08	1.6 ± 0.2*	1.21 ± 0.8	2.3 ± 0.3^++^/^xx^
PC	7.4 ± 0.6	9 ± 1	4 ± 1^#^	1.8 ± 0.3^xxx^
Choline	0.24 ± 0.05	0.40 ± 0.06*	1.5 ± 0.1^###^	1.8 ± 0.3^xx^
TCholine	8.9 ± 0.6	11 ± 1	6 ± 1^#^	5.8 ± 0.5^xx^
Creatine	1.5 ± 0.2	2.4 ± 0.4	6.5 ± 0.6^###^	4.7 ± 0.6^+^/^xx^
PCreatine	1.06 ± 0.08	1.5 ± 0.2*	4 ± 1^#^	2.7 ± 0.8
TCreatine	2.6 ± 0.3	3.9 ± 0.5*	11 ± 1^###^	7.4 ± 0.7^++^/^xx^
Myoinositol	10 ± 1	15 ± 2x	14 ± 2^#^	18 ± 3
Glucose	2.0 ± 0.3	1.7 ± 0.6	0.5 ± 0.2^##^	0.6 ± 0.2
Taurine	1.2 ± 0.3	1.9 ± 0.2*	6.7 ± 0.9^###^	5.5 ± 0.5^xxx^
GSH	3.4 ± 0.4	5.1 ± 0.8*	13 ± 2^###^	6.9 ± 0.9^+^
GSSG	0.7 ± 0.1	0.82 ± 0.08	2.0 ± 0.3^##^	1.1 ± 0.2^+^
TGSH	6.3 ± 0.4	7 ± 1	17 ± 3^##^	10 ± 1^+^/^x^
GSH/GSSG	6 ± 1	7 ± 2	7 ± 1	7 ± 1
ATP	5.5 ± 0.5	6.1 ± 0.4	14 ± 1^###^	9.2 ± 0.8^+^/^x^
Adenosine	1.08 ± 0.07	1.2 ± 0.1	2.4 ± 0.2^###^	2.3 ± 0.1^xxx^
AMP	0.70 ± 0.07	0.62 ± 0.04	1.3 ± 0.5^#^	1.2 ± 0.1^xx^
NADP	0.44 ± 0.05	0.66 ± 0.06*	1.3 ± 0.1^###^	1.20 ± 0.08^xxx^
Glutamate/Glutamine	8 ± 1	7 ± 2	3.3 ± 0.3^###^	5.5 ± 0.7^+^
Glucose/Lactate	0.21 ± 0.05	0.12 ± 0.05	0.04 ± 0.02^##^	0.027 ± 0.008^x^

**TABLE 2:** Lipid metabolite concentration (a.u.) in PFs and PCAFs corresponding to Figure 4. Values were generated from the quantitative analysis of high-resolution ^1^H MR spectra obtained from the aqueous phase of PFs and PCAFs maintained for 48 h under normoxia or under 1% O_2_ (hypoxia). Values represent Mean ± SEM obtained from four to eight independent experiments.

Metabolites	PF normoxia	PF hypoxia	PCAF normoxia	PCAF hypoxia
C**H** = C**H**	12.0 ± 0.6	7.1 ± 0.6 ***	12 ± 2	20 ± 2 ^**++**^/^xxx^
CH = CH-C**H**_**2**_	17.9 ± 0.8	11 ± 1 ***	17 ± 1	29 ± 3 ^+^/^xxx^
(CH = CH-C**H**_**2**_-CH = CH)_n_, n > 1	1.9 ± 0.2	1.20 ± 0.06 **	1.91 ± 0.04	3.0 ± 0.3 ^+^/^xx^
Linoleic acid	3.2 ± 0.2	1.8 ± 0.3 **	4.4 ± 0.3^##^	7.0 ± 0.8 ^+^/^xxx^
Glycerol	3.3 ± 0.2	2.0 ± 0.2 ***	3.2 ± 0.3	5.2 ± 0.5 ^++^/^xxx^
PtdEA	1.5 ± 0.3	1.1 ± 0.1	1.6 ± 0.8	3.5 ± 0.4 ^+^/^xxx^
PtdCho	4.5 ± 0.2	2.8 ± 0.3 ***	4.6 ± 0.4	7.7 ± 0.9 ^+^/^xxx^
Lysophosphatidylcholine	1.1 ± 0.2	0.6 ± 0.2	2.5 ± 0.5^#^	3.7 ± 0.3 ^+^/^xxx^
Sphingomyelin	1.2 ± 0.1	0.8 ± 0.2	1.1 ± 0.2	2.3 ± 0.2 ^++^/^xxx^
Docosahexaenoic acid	0.36 ± 0.02	0.20 ± 0.04 **	0.51 ± 0.01^#^	0.80 ± 0.08 ^+^/^xxx^
AA+ EPA	0.6 ± 0.1	0.3 ± 0.1	1.20 ± 0.08^##^	1.9 ± 0.2 ^+^/^xxx^
Cholesterol	5.0 ± 0.2	3.2 ± 0.2 ***	3.8 ± 0.2^##^	6.5 ± 0.4 ^++^/^xxx^
OO-CH_2_-C**H**_**2**_	18.6 ± 0.9	11.2 ± 0.8 ***	17 ± 1	29 ± 3 ^+^/^xxx^
OOC-C**H**_**2**_	16.1 ± 0.8	9.6 ± 0.8 ***	15 ± 1	26 ± 3 ^+^/^xxx^
Lipids (-C**H**_**2**_-)_n_	243 ± 8	157 ± 11 ***	223 ± 14	376 ± 39 ^+^/^xxx^
Lipids (-C**H**_**3**_)	37 ± 2	23 ± 2 ***	37 ± 3	63 ± 6 ^+^/^xx^

Lipids (-CH_3_): methyl groups of fatty acids, Lipids (-CH_2_-): methylene groups of fatty acids, OOC-C**H**_**2**_-: methylene groups at the α position of the carboxylic function, OOC-CH_2_-C**H**_**2**_-: methylene groups at the β position of the carboxylic function, ARA: arachidonic acid, EPA: eicosapentaenoic acid, PtdEA: phosphatidylethanolamine, PtdCholine: phosphatidylcholine, (CH = CH-C**H**_**2**_-CH = CH)n: diallylic methylene protons, CH = CH-C**H**_**2**_-: methylene groups at the α position of a double bond, CH = CH: fatty acid double bonds. *p < 0.05, **p < 0.01, ***p < 0.001 PF normoxia vs PF hypoxia; ^+^ *p* < 0.05, ^++^ *p* < 0.01, ^+++^ *p* < 0.001 PCAF normoxia vs PCAF hypoxia; ^#^ *p* < 0.05, ^##^ *p* < 0.01, ^###^ *p* < 0.001 PF normoxia vs PCAF normoxia; ^x^ *p* < 0.05, ^xx^ *p* < 0.01, ^xxx^ *p* < 0.001 PF hypoxia vs PCAF hypoxia.

As evident from the spectra, figures and tables, the metabolic response to hypoxia was significantly different in PFs and PCAFs. 10 metabolites were commonly altered in PFs and PCAFs with hypoxia, but the changes were in opposite directions. Glutamine, aspartate, GPC, and glutathione increased in PFs and decreased in PCAFs under hypoxia. Linoleic acid, glycerol, phosphatidyly-N-trimethylethanolamine, lysophosphatidylcholine, docosahexanoic acid and cholesterol decreased in PFs and increased in PCAFs under hypoxia. Outside of the common metabolites, in PFs glutamate, choline, phosphocreatine, myoinositol, taurine and NADP increased with hypoxia. In PCAFs, arginine, alanine, pyruvate, lactate, creatine, oxidized glutathione, and ATP decreased with hypoxia and 3-phosphatidylethanolamine, sphingomyelin and arachidonic acid increased with hypoxia.

Several metabolites that were significantly different between PFs and PCAFs under normoxia were also significantly different between PFs and PCAFs under hypoxia with the exception of cholesterol that decreased in PCAFs under normoxia and increased under hypoxia. Glutamine, glutamate, arginine, alanine, pyruvate, creatine, lactate, phosphocholine, choline, taurine, ATP, adenosine, NADP, linoleic acid, sphingomyelin, docosahexaenoic acid, arachidonic acid and cholesterol were significantly higher in PCAFs compared to PFs under normoxia as well as under hypoxia. Outside of these common metabolites, under normoxia, aspartate, phosphocreatine, myoinositol, glutathione, and oxidized glutathione were significantly higher in PCAFs compared to PFs, and glucose was significantly lower in PCAFs compared to PFs. Under hypoxia, GPC, AMP, glycerol, 3-phosphatidylethanolamine, phosphatidyl-N-trimethylethanolamine and lysophosphatidylcholine were higher in PCAFs compared to PFs.

Multiple pathways were altered in PFs and PCAFs under normoxia and hypoxia. Five pathways were identified as commonly altered in both PFs and PCAFs in response to hypoxia using the ConsensusPathDB analysis. Seven altered pathways were uniquely altered in PFs when comparing PFs under hypoxia to PFs under normoxia, and twelve pathways were uniquely altered in PCAFs when comparing PCAFs under hypoxia to PCAFs under normoxia. Pathways unique to PFs included biosynthesis of protectin and resolvin conjugates in tissue regeneration, biosynthesis of DHA-derived sulfido conjugates, glycerophospholipid catabolism, pathways involved in maresin biosynthesis, and glutamate neurotransmitter release cycle. Pathways unique to PCAFs included ABC transporters in lipid homeostasis, phospho-PLA2 pathway, Ca-dependent events, effects of PIP2 hydrolysis, alanine metabolism, proton-coupled monocarboxylate transport, platelet sensitization by LDL, regulation of lipid metabolism by PPAR alpha, hedgehog ligand biogenesis, cellular response to starvation, arachidonate production from DAG, and conjugation of phenylacetate with glutamine.

When comparing PFs to PCAFs under normoxia and hypoxia, eleven pathways were uniquely altered under normoxia, thirty pathways were common to normoxia and hypoxia, and thirty-eight pathways were uniquely altered under hypoxia.

### PD-L1 expression in PFs and PCAFs

**FIGURE 6:**
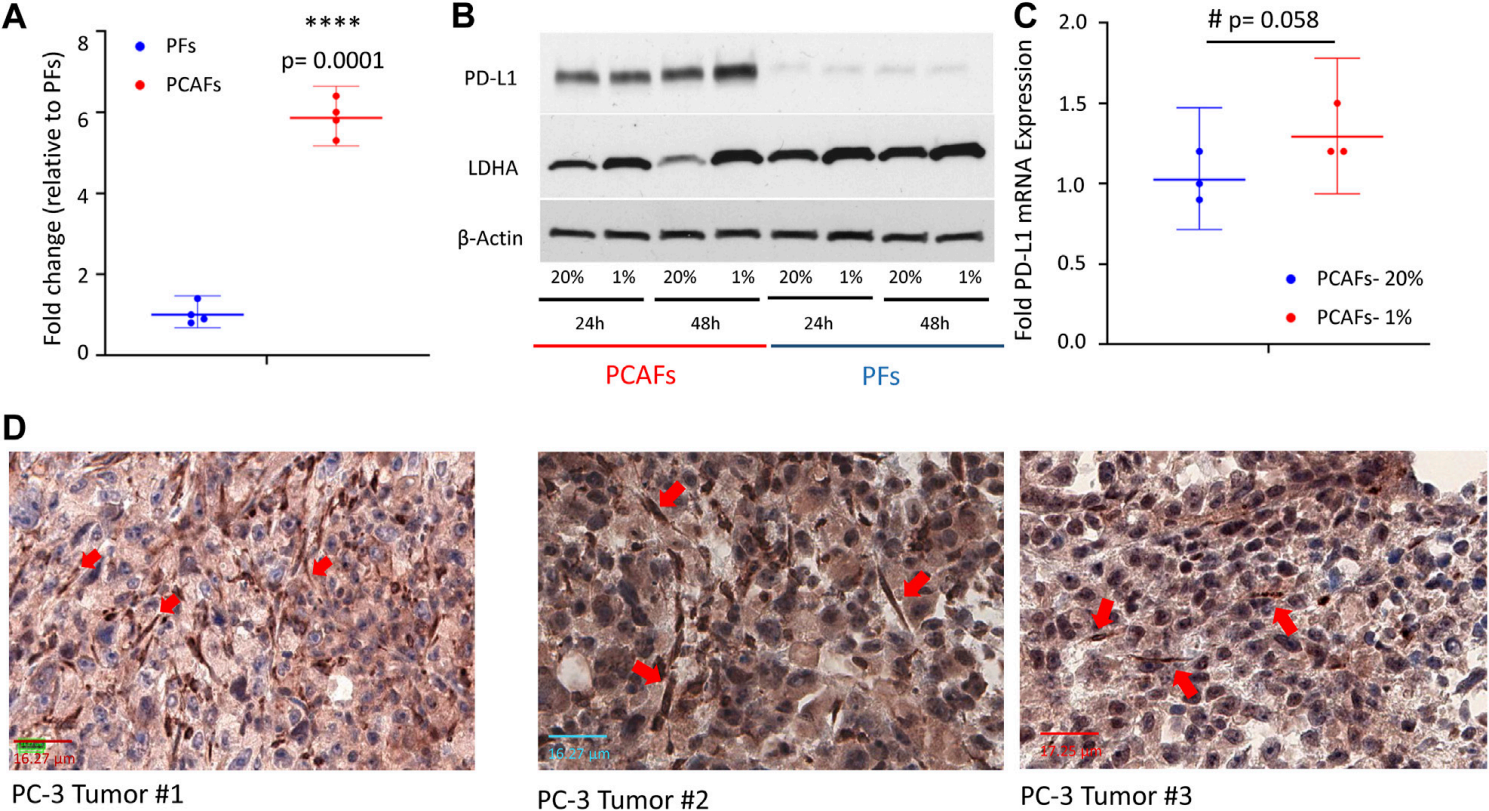
**(A)** PD-L1 mRNA was significantly higher in PCAFs compared to PFs under normoxia. Fold change were normalized to PF levels. Values are Mean ± SE, n = 4. **(B)** PD-L1 immunoblots of PCAFs and PFs under normoxia and hypoxia. PCAFs show elevated PD-L1 compared to PFs. At 48 h hypoxia increased PCAF PD-L1 but not PF PD-L1; increased LDHA is used as a hypoxia marker. **(C)** PD-L1 mRNA increased with hypoxia in PCAFs but not in PFs. **(D)** Representative PD-L1 immunostained sections from three PC-3 hPCa xenografts showing increased PD-L1 expression by CAFs (red arrows).

**FIGURE 7:**
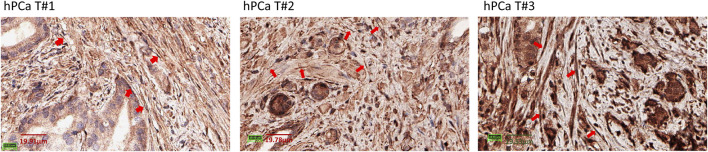
PD-L1 immunostaining of hPCa tissue. Reprentative ×40 magnification images of 3 different hPCa tissue samples showing increased PD-L1 expression by CAFs (red arrows). hPCa T#1: T2N0M0, grade 2, stage II; hPCa T#2: T2N0M0, grade 3, stage II; hPCa T#3: T3N0M0, grade 2–3, stage III.

We characterized PD-L1 expression in PFs and PCAFs under normoxia and hypoxia as shown in Figures 6A–C. PD-L1 mRNA was significantly higher in PCAFs compared to PFs under normoxia (Figure 6A) that was confirmed in the immunoblots of these cells under normoxia as shown in Figure 6B. Under hypoxia, we observed a significant increase of PD-L1 mRNA in PCAFs, but not in PFs (results not shown), that was confirmed by an increase of PD-L1 protein in PCAFs, but not PFs, under hypoxia. Representative tumor sections from three different PC-3 hPCa xenografts immunostained for PD-L1 are presented in Figure 6D that further confirm the expression of PD-L1 by PCAFs in the tumor section. Further, as presented in Figure 7, PD-L1 immunostaining of hPCa tissue identified expression of PD-L1 by PCAFs.

## Discussion

Our data identified clear differences between PFs and PCAFs under normoxia and in their response to hypoxia. These differences were identified in ECM degradation and invasion, metabolism and PD-L1 expression.

Our observations that PFs were invasive and degraded ECM under normoxia, and that invasion and degradation increased under hypoxia are consistent with the roles of fibroblasts in wound healing (Jiang et al., 2023). PCAFs, on the other hand were less invasive and less able to degrade ECM under normoxia. PCAFs became even less invasive with a significant reduction of ECM degradation under hypoxia. Our results are in agreement with recent studies that suggest that hypoxia suppresses the pro-tumorigenic remodeling of the tumor microenvironment by CAFs, thereby inhibiting tumor growth and metastasis (Kim et al., 2022). Hypoxia has been found to activate TIMP metallopeptidase inhibitor 1 (TIMP1), suppressing matrix metalloproteinases (MMPs) and ECM turnover in renal fibroblasts (Norman et al., 2000). Taken together, our results suggest that normal fibroblasts, located at the tumor periphery, may assist with cancer cell invasion and metastasis by degrading the ECM, while PCAFs are not directly involved in ECM degradation. Nevertheless, PCAFs may play a role by inducing other cells to degrade ECM through the expression of several cytokines (Orimo et al., 2005; Grugan et al., 2010) and by remodeling the ECM (Gaggioli et al., 2007; Goetz et al., 2011) under normoxia and under hypoxia.

PFs and PCAFs displayed significantly different metabolic profiles under normoxia and under hypoxia. Under normoxia, PCAFs showed lower glucose, in good agreement with previous studies that have reported a shift to aerobic glycolysis in CAFs (Martinez-Outschoorn et al., 2011; Zhang et al., 2015). High levels of energy rich metabolites such as lactate and pyruvate were found in PCAFs, in good agreement with previous results in breast and bladder CAFs (Whitaker-Menezes et al., 2011; Shi et al., 2015). These differences are consistent with the so-called “reverse Warburg hypothesis” (Pavlides et al., 2009; Gonzalez et al., 2014) that suggests that tumor metabolism consists of two coupled metabolic compartments containing anabolic cancer cells and catabolic CAFs (Avagliano et al., 2018). In this model, cancer cells do not rely on glucose oxidation, but rather utilize lactate and pyruvate produced by CAFs to support several processes such as the tricarboxylic acid cycle, anabolic processes, and cell proliferation (Li et al., 2021). These changes are coupled with cancer cells inducing increased expression of glucose transporter 1 (GLUT1) and monocarboxylate transporter 4 (MCT4) in PCAFs, increasing glucose uptake and lactate output by these fibroblasts (Fiaschi et al., 2012). CAF derived lactate may also have other functions such as maintaining increased mitochondrial activity and oxidative phosphorylation in PCa cells (Ippolito et al., 2019), and activating the TGF-β1/p38 MAPK/MMP2/9 pathway to promote invasion in breast cancer cells (Sun et al., 2019).

PCAFs also displayed higher levels of different amino acids such as glutamine, glutamate, aspartate, arginine and alanine, consistent with the possibility that CAFs assist cancer cells to withstand nutrient deprivation (Olivares et al., 2017), thereby promoting tumor growth and development (Orimo et al., 2005; Li et al., 2021). Transport of amino acids from CAFs to cancer cells has been observed in ovarian cancer (Yang et al., 2016), human skin squamous carcinoma (Bertero et al., 2019), and in prostate and pancreatic cancer (Sousa et al., 2016; Zhao et al., 2016). PCAFs also contained lower levels of total choline and PC compared with PFs, suggesting that the high levels or choline containing compounds found in many solid tumors are primarily due to cancer cells (Glunde et al., 2005; Mariotto et al., 2018). PCAFs showed higher levels of GSH, GSSG and total glutathione, although the ratio of reduced to oxidized glutathione was similar to PFs. This is in good agreement with previous studies reporting on the role of CAFs in counteracting drug-induced oxidative stress by increasing the levels of GSH in pancreatic tumor cells (Cheteh et al., 2017).

Unlike the aqueous phase metabolites, metabolites detected in the lipid phase of PFs and PCAFs were similar, although previous studies have identified increased CAF lipid production in colorectal (Gong et al., 2020) and pancreatic cancer (Auciello et al., 2019). Changes in lipids in response to hypoxia contrasted in PFs and PCAFs with the lipid content in PFs significantly reduced with hypoxia and in PCAFs significantly increased with hypoxia. The PCAF data are in agreement with previous studies reporting that lipid metabolism and lipid droplet accumulation increased under hypoxia (Laurenti et al., 2011; Shen et al., 2012). Increased lipid production by CAFs may make more lipids available to cancer cells (Gong et al., 2020) to support cancer cell growth and the synthesis of new lipids (Li et al., 2021).

Multiple metabolites were altered in common in PFs and PCAFs in response to hypoxia, but these changes were in opposite directions highlighting the metabolic reprogramming that occurred in the PCAFs investigated here. Multiple molecular pathways associated with these metabolites were also identified in the hypoxic response but based on the opposing directions of the metabolic changes, the alterations in the pathways were also likely to have been in opposite directions. Future studies should investigate the upregulation or downregulation of these pathways.

PCAFs displayed an immunosuppressive profile, as the production of metabolites such as lactate (Feng et al., 2017), glutamate (Xia et al., 2018), or glutamine (Wang et al., 2018) have been linked to increased immune resistance of tumors. Furthermore, it has been shown that lipids can reprogram tumor infiltrating myeloid and T cells towards immunosuppressive and anti-inflammatory phenotypes (Herber et al., 2010; Al-Khami et al., 2017; Muroski et al., 2017; Niu et al., 2017), reinforcing the immune suppressive role of CAFs. We found significantly increased levels of PD-L1 in PCAFs compared to PFs, supporting their immune suppressive profile. PD-L1 expression by CAFs was also observed in human PCa xenografts, consistent with earlier studies (Brennen et al., 2016; Brennen et al., 2021). Under hypoxia, PD-L1 increased further in PCAFs but decreased in PFs, further supporting the role of PCAFs in creating an immune suppressive microenvironment in hypoxic tumor regions. The increase of PD-L1 under hypoxia in PCAFs is consistent with our previous study demonstrating the hypoxic regulation of PD-L1 expression in human breast cancer cells and tumor models (Parkins et al., 2023).

In summary, our results expand our understanding of the reprogramming that can occur in PCAFs that modifies their invasion, metabolism and immune checkpoint expression under normoxia and hypoxia compared to PFs. PCAFs were less able to invade and degrade the ECM especially under hypoxia, that may reduce invasion and metastasis, but displayed an immunosuppressive metabolic profile as well as increased expression of PD-L1 under hypoxia that may contribute to escaped immune surveillance. Future expanded studies with a panel of PFs and PCAFs should further validate these reprogrammed differences. While here we focused on PD-L1, future studies should focus on investigating the effects of hypoxia on known immune checkpoints in PFs and PCAFs. Additional studies with subtypes of PCAFs (Pan et al., 2023) will further expand our understanding of the role of CAFs in PCa. Our data identify potential metabolic and molecular pathway-based targets for PCAF-based therapies to prevent CAFs from assisting PCa cells in progression and dissemination under normoxia and hypoxia.

## Data Availability

The raw data supporting the conclusion of this article will be made available by the authors, without undue reservation.

## Abbreviations

BBI, Broad band inverse; CAFs, Cancer-associated fibroblasts; DW, Diffusion weighted; ECM, Extracellular matrix; FBS, Fetal bovine serum; FOV, Field of view; GLUT1, Glucose transporter 1; GSH, Reduced glutathione; GSSG, Oxidized glutathione; hPCa, Human prostate cancer; HPRT1, Hypoxanthine phosphoribosyltransferase 1; HR, High resolution; LDHA, Lactate dehydrogenase A; MCT4, Monocarboxylate transporter 4; MMP, Matrix metalloproteinase; MRI, Magnetic resonance imaging; MRS, Magnetic resonance spectroscopy; PCa, Prostate cancer; PCAF, Prostate cancer associated fibroblast; PF, Prostate fibroblast; PD-L1, Programmed death ligand-1; RIPA, Radio-immunoprecipitation assay; ROI, Region of Interest; TE, Echo Time; TMA, Tissue microarray; TME, Tumor microenvironment; TMS, Tetramethylsilane; TR, Repetition Time; TSP, 3-(trimethylsilyl) propionic 2,2,3,3-d4 acid sodium salt.
