# Supplementary figures and images for: Prostate fibroblasts and prostate cancer associated fibroblasts exhibit different metabolic, matrix degradation and PD-L1 expression responses to hypoxia
Source: Front Mol Biosci. 2024 Mar 22;11:1354076. doi: 10.3389/fmolb.2024.1354076 (PMC10995317; doi:10.3389/fmolb.2024.1354076)

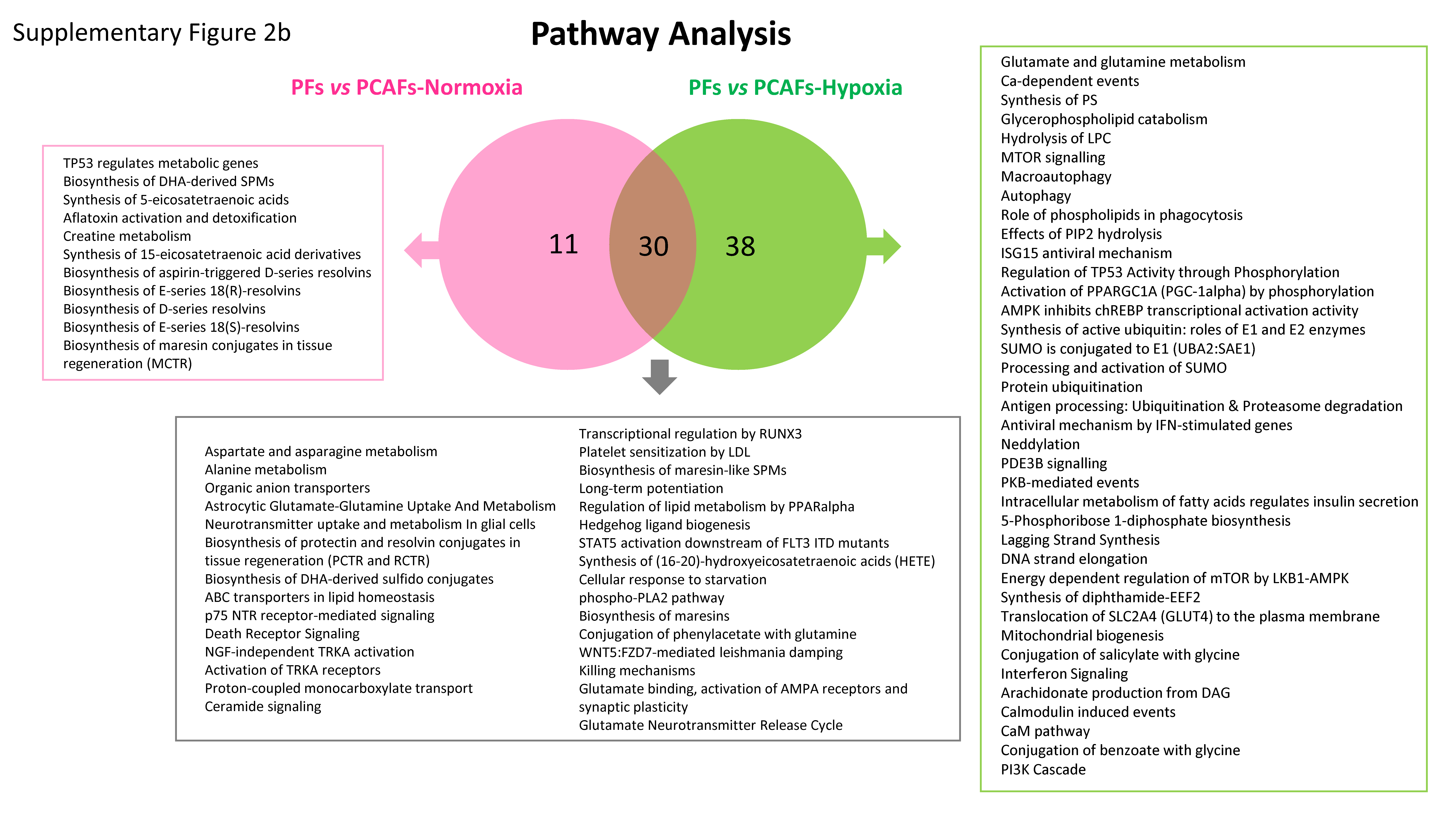

Supplement: Supplementary file 1 [file Image3.TIFF]

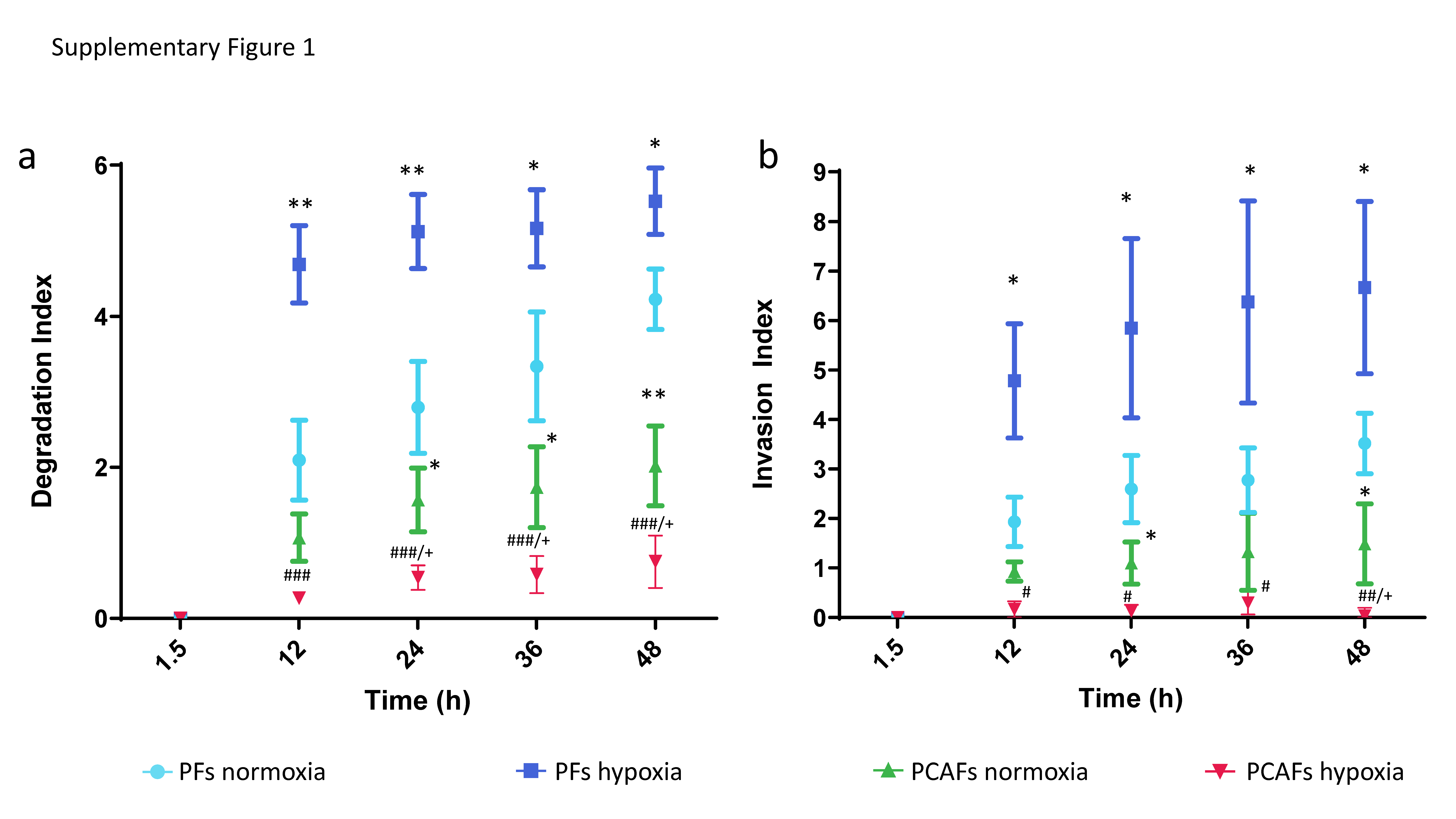

Supplement: Supplementary file 2 [file Image1.TIFF]

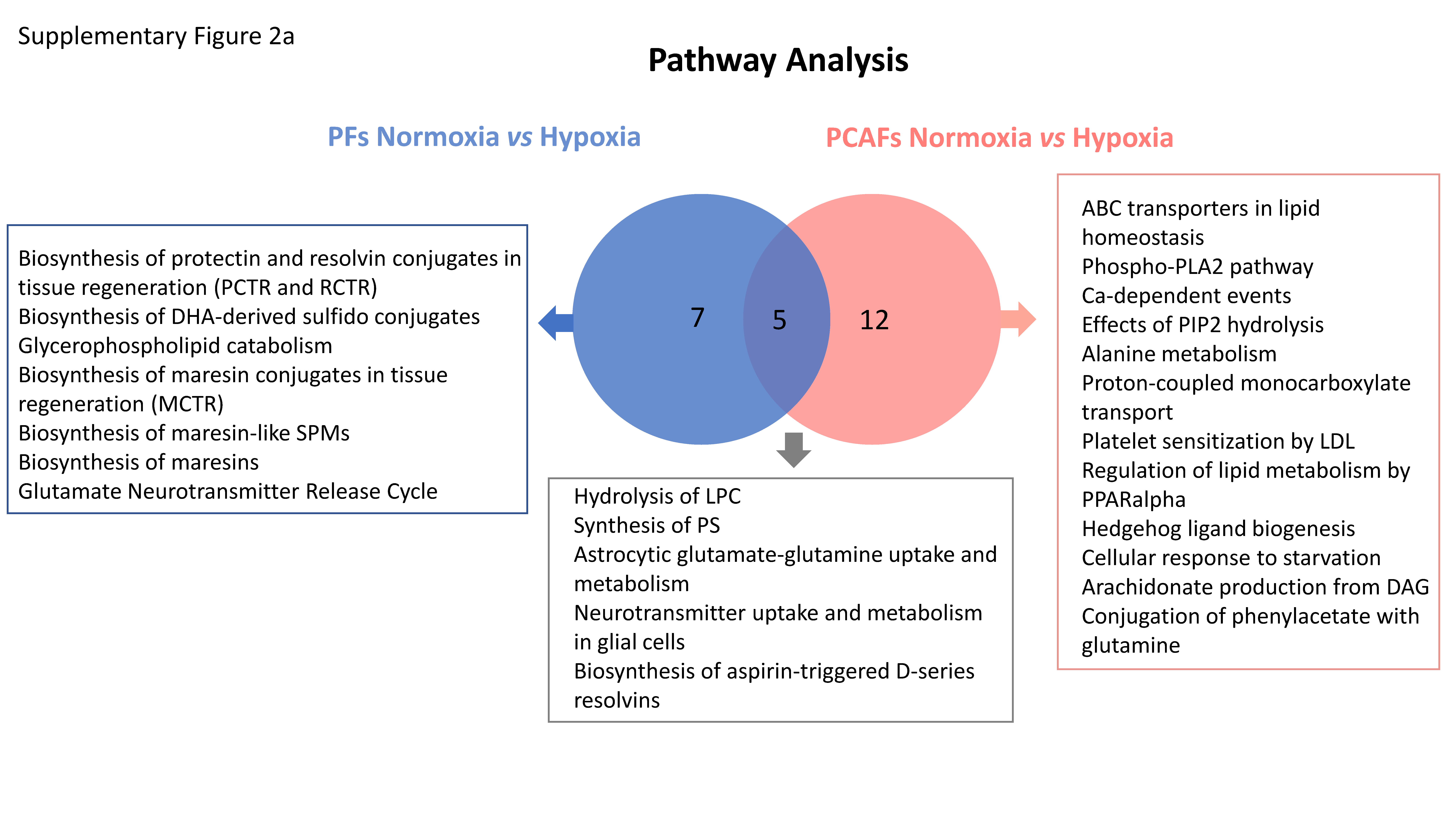

Supplement: Supplementary file 3 [file Image2.TIFF]
